# An anti-inflammatory diet as treatment for inflammatory bowel disease: a case series report 

**DOI:** 10.1186/1475-2891-13-5

**Published:** 2014-01-16

**Authors:** Barbara C Olendzki, Taryn D Silverstein, Gioia M Persuitte, Yunsheng Ma, Katherine R Baldwin, David Cave

**Affiliations:** 1 Division of Preventive and Behavioral Medicine, University of Massachusetts Medical School, 55 Lake Ave North, Shaw Building, Worcester MA, USA; 2 Department of Gastroenterology, (UMass) Memorial Medical Center, 55 Lake Ave North, Worcester MA, USA; 3 Department of Medicine and Pediatrics, UMass Memorial Medical Center, Worcester MA, USA

**Keywords:** Diet, Inflammatory bowel disease, Nutrition

## Abstract

**Background:**

The Anti-Inflammatory Diet (IBD-AID) is a nutritional regimen for inflammatory bowel disease (IBD) that restricts the intake of certain carbohydrates, includes the ingestion of pre- and probiotic foods, and modifies dietary fatty acids to demonstrate the potential of an adjunct dietary therapy for the treatment of IBD.

**Methods:**

Forty patients with IBD were consecutively offered the IBD-AID to help treat their disease, and were retrospectively reviewed. Medical records of 11 of those patients underwent further review to determine changes in the Harvey Bradshaw Index (HBI) or Modified Truelove and Witts Severity Index (MTLWSI), before and after the diet.

**Results:**

Of the 40 patients with IBD, 13 patients chose not to attempt the diet (33%). Twenty-four patients had either a good or very good response after reaching compliance (60%), and 3 patients’ results were mixed (7%). Of those 11 adult patients who underwent further medical record review, 8 with CD, and 3 with UC, the age range was 19–70 years, and they followed the diet for 4 or more weeks. After following the IBD-AID, all (100%) patients were able to discontinue at least one of their prior IBD medications, and all patients had symptom reduction including bowel frequency. The mean baseline HBI was 11 (range 1–20), and the mean follow-up score was 1.5 (range 0–3). The mean baseline MTLWSI was 7 (range 6–8), and the mean follow-up score was 0. The average decrease in the HBI was 9.5 and the average decrease in the MTLWSI was 7.

**Conclusion:**

This case series indicates potential for the IBD-AID as an adjunct dietary therapy for the treatment of IBD. A randomized clinical trial is warranted.

## Introduction

Crohn’s disease (CD) and ulcerative colitis (UC) are two chronic inflammatory diseases of the gastrointestinal tract, collectively known as inflammatory bowel disease (IBD). Each is distinct in its presentation and course of disease, and both are chronically relapsing illnesses. Despite histological differences between the two diseases, dietary therapy (with the exception of known efficacy of enteral therapy and CD) has often been similar for both [1], involving options that include increased fluids, avoidance of fatty foods, dietary fiber, and dairy products if lactose intolerant. Others have ingested nothing by mouth for a period of time, and used tube feeding with elemental diets [2]. Presently, an alteration of the intestinal microbiome is believed to be a major factor in the pathogenesis of IBD, and this change provides a possible pathway for dietary manipulations of the microbiome that may reduce inflammation in these conditions [3,4].

The goals for treatment of IBD are the induction of remission, maintenance of remission, reduction in the need for long-term use of corticosteroids, improved quality of life, and improved prognosis. The mainstays of current treatment comprise of anti-inflammatory agents including corticosteroids, immunomodulators, and biologic agents. There are surgical options including colectomy, which while curative for patients with UC, can result in a pouch or end ileostomy in approximately 30% of patients [5]. Neither of these surgical options is without complications and up to 70% of patients with a pouch may develop pouchitis [6,7]. An even higher percentage of CD patients need surgery, with upward of 80% of patients requiring surgical resection that is not necessarily curative [5].

Until recently, the investigation of nutritional approaches in treating the diseases have been largely limited to the use of enteral and total parenteral nutrition with the aim of providing bowel rest [8,9]. Dietary whole food recommendations for CD and UC are poorly developed, even though patients often ask for advice with their diet [10]. To prepare clinicians for the complex management of patients with IBD, Brown et al. published dietary guidelines to fill this gap, [2] compiling a summary of suggestions from reputable organizations, concluding that no specific diet currently exists for patients with IBD. Most dietary guidelines, though varied in scope and complexity, indicate that limiting lactose, excess fat, excess carbohydrates, and reducing fiber in the diet is necessary, particularly during flares of disease [2]. Mineral and vitamin supplementation may or may not assist with nutrient deficiencies and malabsorption. In addition, many patients also experience other food intolerances [11,12]. To provide a dietary therapy approach addressing nutrient adequacy, malabsorption issues, and symptoms, we developed the IBD-Anti-Inflammatory Diet, or IBD-AID [13-15]. The diet was offered to patients who were refractory to pharmacological therapy, or treatment was not as effective as desired. This is a retrospective case series to demonstrate the beneficial effects of the IBD-AID on a small number of patients.

## Materials and methods

### Patients

Forty patients received instructions for the diet, 37 saw the nutritionist, and 3 were seen only by gastroenterologists. Medical records review was approved by the Institutional Review Board (IRB) of the University of Massachusetts Medical School. Eligibility for this further review included seeing an IRB-approved gastroenterologist and dietitian in clinic, endoscopically-diagnosed IBD and failure of drug treatment, persistent symptoms, or reluctance to proceed with other options (Table 1). Medical records of the eleven patients for whom we had complete data and used the IBD-AID to help treat their disease were retrospectively reviewed and their progress was assessed using the Harvey Bradshaw (HBI) and Modified Truelove and Witts Severity Index (MTLWSI) scoring systems [16,17] applied before and during the use of the IBD-AID. Review of these patients included clinic notes from gastroenterologists, primary care physicians, nutritionists, surgeons, hospital admission and discharge summaries, as well as laboratory data (i.e.; hematocrit, albumin, erythrocyte sedimentation rate (ESR), C-reactive protein (CRP), drug concentration levels, imaging studies, and endoscopic evaluation. An HBI for CD patients or MTLWSI for UC patients was included or estimated from information provided by clinic notes. The data was compared before dietary treatment, and evaluated 1–3 months after patients stated they followed the IBD-AID.

**Table 1:** Participant demographics, medications, and disease indices

**Age**	**Sex**	**Disease**	**Prior treatment includes**	**Recent treatment**	**HBI/MTLWSI before**		**HBI/MTLWSI after**
39	F	CD	ASA, IM, B	ASA?+?IBD-AID	HBI	12	3
47	F	CD	S, IM, B	S(taper)?+?IBD-AID	HBI	9	2
39	F	CD	S,IM	IM?+?IBD-AID	HBI	12	2
24	F	CD	S,ASA, IM, B	S(taper) IM?+?IBD-AID	HBI	15	0
39	M	CD	IM, B	IBD?+?AID	HBI	20	0
40	M	CD	S,ASA, IM	IM?+?IBD-AID	HBI	15	2
41	M	CD	ASA, IM	IM?+?IBD-AID	HBI	4	2
37	F	CD	S,ASA, B, elemental diet	B?+?IBD-AID	HBI	1	1; histologic remission
69	M	UC	ASA, IM, B	ASA, IM?+?IBD-AID	MTLWSI n/d		2; improved
19	F	UC	S, ASA, IM, B	ASA, IBD-AID	MTLWSI 6		0
70	F	UC	ASA, IM, B	B?+?IBD-AID	MTLWSI 8		0

Legend:

ASA?=?5-Aminosalycylic acid agent.

IM?=?Immunomodulator.

B?=?Biologic.

S?=?Steroid.

IBD-AID?=?Inflammatory Bowel Disease-Anti-Inflammatory Diet.

HBI?=?Harvey Bradshaw Index.

MTLWSISI?=?Modified TrueLove and Witts Severity Index.

### The diet

The goal of the IBD-AID is to assist with a decreased frequency and severity of flares, obtain and maintain remission. Dysbiosis, or altered bacterial flora, is one of the theories behind the development of IBD-AID, in that certain carbohydrates in the lumen of the gut provide pathogenic bacteria a substrate on which to proliferate [17-21]. The IBD-AID has five basic components: the first of which is the modification of certain carbohydrates, (including lactose, and refined or processed complex carbohydrates) the second places strong emphasis on the ingestion of pre- and probiotics (e.g.; soluble fiber, leeks, onions, and fermented foods) to help restore the balance of the intestinal flora [22-25], and the third distinguishes between saturated, trans, mono- and polyunsaturated fats [26-29], the fourth encourages a review of the overall dietary pattern, detection of missing nutrients, and identification of intolerances. The last component modifies the textures of the foods (e.g.; blenderized, ground, or cooked) as needed (per patient symptomology, see Table 2) to improve absorption of nutrients and minimize intact fiber. The phases indicated in Table 2 are examples of the modification of texture complexity, so that dietitian and patient can expand the diet as the patient’s tolerance and absorption improves. Some sensitivities common to many patients (not just those with IBD), are eased through supplementation of digestive enzymes or avoidance. A senior dietitian advised the patient and either family or spouse regarding the details of the diet during regular clinic visits. Patients taking supplements (probiotics, vitamin/minerals, omega-3 fatty acids) were advised to continue or discontinue, depending on the needs of the individual and the dietary intake.

**Table 2:** IBD-AID food phase chart

**Phase type**	**Phase I**	**Phase II**	**Phase III**	**Phase IV**
	***Soft, well-cooked or cooked then pureed foods, no seeds***	***Soft Textures: well-cooked or pureed foods, no seeds, choose floppy or tender foods***	***May still need to avoid stems, choose floppy greens or other greens depending on individual tolerance***	***If in remission with no strictures***
Vegetables	Butternut Squash, Pumpkin, Sweet Potatoes, Onions	Carrots, Zucchini, Eggplant, Peas, Snow peas, Spaghetti squash, Green beans, Yellow beans, Microgreens (2 week old baby greens), Watercress, Arugula, Fresh flat leaf parsley and cilantro, Seaweed, Algae	Butter lettuce, Baby spinach, Peeled cucumber, Olives, Leeks Bok Choy, Bamboo shoots, Collard greens, Beet greens, Sweet peppers, Kale, Fennel bulb	Artichokes, Asparagus, Tomatoes, Lettuce, Brussels sprouts, Beets, Cabbage, Kohlrabi, Rhubarb, Pickles, Spring onions, Water chestnuts, Celery, Celeriac, Cauliflower, Broccoli, Radish, Green pepper, Hot pepper
	Pureed vegetables: Mushrooms, Phase II vegetables (pureed)	Pureed vegetables: all except cruciferous	Pureed vegetables: all from Phase IV, Kimchi	
Fruits	Banana, Papaya, Avocado, Pawpaw	Watermelon (seedless), Mangoes, Honeydew, Cantaloupe, May need to be cooked: Peaches, Plums, Nectarines, Pears, (Phase III fruits are allowed if pureed and seeds are strained out)	Strawberries, Cranberries, Blueberries, Apricots, Cherries, Coconut, Lemons, Limes, Kiwi, Passion fruit, Blackberries, Raspberries, Pomegranate (May need to strain seeds from berries)	Grapes, Grapefruit, Oranges, Currants, Figs, Dates, Apples (best cooked), Pineapple, Prunes
Meats and fish	All fish (no bones), Sardines (small bones ok), Turkey and ground beef, Chicken, Eggs	Scallops	Lean cuts of Beef, Lamb, Duck, Goose	Shrimp, Prawns, Lobster
Non dairy unsweetened	Coconut milk, Almond milk, Oat milk, Soy milk			
Dairy, unsweetened	Yogurt, Kefir	Farmers cheese (dry curd cottage cheese), Cheddar cheese	Aged cheeses	
Nuts/Oils/Legumes/Fats	Miso (refrigerated), Tofu, Olive oil, Canola oil, Flax oil, Hemp oil, Walnut oil, Coconut oil	Almond flour, Peanut flour, Soy flour, Sesame oil, Grapeseed oil, Walnut oil, Pureed nuts, Safflower oil, Sunflower oil	Whole nuts, Soybeans, Bean flours, Nut butters, Well-cooked lentils (pureed), Bean purees (e.g. hummus)	Whole beans and lentils
Grains	Ground flax or Chia Seeds (as tolerated)	Steel cut oats (well-cooked as oatmeal)	Rolled well-cooked oats	
Spices	Basil, Sage, Oregano, Salt, Nutmeg, Cumin, Cinnamon, Turmeric, Saffron, Mint, Bay leaves, Tamari (wheat free soy sauce), Fenugreek tea, Fennel tea, Vanilla	Dill, Thyme, Rosemary Tarragon, Cilantro, Basil, Parsley	Mint, Ginger, Garlic (minced), Paprika, Chives, Daikon, Mustard	Wasabi, Tamarind, Horseradish, Fenugreek, Fennel
Sweeteners	Stevia, Maple syrup, Honey (local), Unsweetened fruit juice	Lemon and lime juice		
Misc.	Capsule or liquid supplements, Cocoa powder	Baking powder (no cornstarch), Baking soda, Unflavored gelatin	Ghee, Light mayonnaise, Vinegar	Ketchup (sugar free), Hot sauce (sugar free)

The IBD-AID consists of lean meats, poultry, fish, omega-3 eggs, particular sources of carbohydrate, select fruits and vegetables, nut and legume flours, limited aged cheeses (made with active cultures and enzymes), fresh cultured yogurt, kefir, miso and other cultured products (rich with certain probiotics) and honey. Prebiotics, in the form of soluble fiber (containing beta-glucans and inulin, such as bananas, oats, blended chicory root, and flax meal) are suggested. In addition, the patient is advised to begin at a texture phase of the diet matching with symptomology, starting with phase one if in an active flare. Many patients require foods to be softened and textures mechanically altered by pureeing the foods, and avoiding foods with stems and seeds when starting the diet (see phases 1–3 of Table 2), as intact fiber can be problematic for those with strictures and highly active mucosal inflammation. Some patients will require lifelong avoidance of intact fiber. Food irritants are not limited to intact fiber, but may include certain foods, processing agents and flavorings to which IBD patients may be reactive.

For the purposes of this study, compliance was determined by food records (patient self-report), and included all food components for the duration of the study. After the first visit with the dietitian, patients were asked to keep detailed food records, along with time and recording of symptoms (on a scale of 0–5), with follow-up appointments occurring 2–3 weeks after the initial consult. Symptoms included a subjective assessment of bloating, pain, diarrhea, urgency, bleeding, and fatigue. Other influences to health that were recorded by patients included perceived stress, and origin of food intake. Most patients indicated difficulty in understanding and adhering to the diet initially, while tracking trends of symptomatic progress. The form and type of foods recommended in the beginning were based on the severity of symptoms reported, with the goal to deliver nutrients even if the patient started the diet while in a flare. As symptoms improved, patients were advanced to more whole foods, but within the guidelines of the IBD-AID. One of the patients did not improve within the first 2–3 weeks, and the diet was reviewed for foods of intolerance. In this case, the patient who continued to experience symptoms (loose bowels, dyspepsia and bloating) improved through stronger adherence and changing the texture/form of foods consumed. All patients were then advanced cautiously as symptoms improved.

### Clinical assessment

The HBI was used to assess study patients with CD. The HBI is a simplified, less cumbersome alternative to the Crohn’s Disease Activity Index (CDAI), and is designed to make data collection and calculation easier, with greater application to clinical practice since it is based on the last 24 hours of data [17]. A prospective study of 112 patients with Crohn’s disease showed that the CDAI and HBI scores were highly correlated (r?=?0.93) [17]. Our analyses define HBI remission as an HBI score of less than or equal to 4 points, and HBI response as a decrease in HBI score of greater than or equal to 3 points. The MTLWSI was used for patients with UC. First described in 1990 by Lichtiger and colleagues [16] in a trial for the treatment of UC, the MTLWSI includes a scoring system ranging from 0–21 points, and clinical response is defined as a decrease from the baseline score of 50%, or greater or less than 10 on 2 consecutive days. Clinical remission criteria was determined by a MTWSI of less than or equal to 2, and clinical response was defined as a reduction from baseline in the MTWSI of greater than or equal to 2 points [30].

## Results

Out of the original 40 patients, 37 were seen by the dietitian. Of those, 13 did not attend any follow-up visits. Three had an ambivalent or negative response to the diet, and 24 had a good or very good response to the diet, as measured by self-report of symptoms and compliance with the diet through food records. Of the patients reporting improvement, they were all able to reach greater than 70% compliance to the diet. Of the 3 patients (2 with UC, 1 with CD) with ambivalent or negative response, 2 were diagnosed with Clostridium Difficile (C-diff), and the other (UC) patient’s response is unattributable.

The 11 patients, who met eligibility by IRB and had complete data, underwent medical record review: 8 have CD, 3 have UC, and age range was 19–70 years (see Table 1). All patients used the diet for at least 4 weeks. Before the dietary intervention, 7 patients (64%) had 1 or more drug treatment failures, meaning they had adverse side effects, the medication had no effect, or the medication had become ineffective over time. After using the IBD-AID, which was the only additional intervention added, all (100%) of the patients worked with their gastroenterologists to downscale their medication regimen and all (100%) of the patients had their IBD symptoms reduced. Of the CD patients, the baseline HBI averaged 11 (range 1 to 20), and after dietary intervention, the HBI averaged 1.5 (range 0 to 3). The UC patients had a mean baseline score of MTLWSI of 7 (range 6 to 8, with one patient unable to report baseline), and their mean follow-up score was 0. The mean decrease in the HBI was 9.5 and the mean decrease in the MTLWSI was 7 (Table 1).

## Discussion

Our data suggest that at least some of our patients with inflammatory bowel disease can benefit from the use of the IBD-AID, particularly in terms of reducing symptomatology and consequently a reduction in the use of medication. A portion (33%) of patients who were offered the diet declined initiation, and of those who decided to participate, many encountered behavioral and intellectual barriers that are to be expected with any comprehensive change in dietary lifestyle. The assistance of a partner also trained in the IBD-AID was clearly beneficial [31]. In the current study, due to small numbers of patients and the case series study design, we were unable to confirm our hypothesized mechanisms of action by examining the impact of the IBD-AID on the intestinal microbiome. However, as deep sequencing and other techniques for defining the microbiome become less expensive this may prove an objective measure of the benefit not only of the IBD-AID, but other diets as well; particularly if the microbiome can be shown to normalize as mucosal healing occurs.

Studies have suggested that IBD results from an inappropriate activation of the mucosal immune system in a genetically susceptible host [30]. The intraluminal gut flora appears to play a role in the inflammatory response and pathogenesis of IBD [32]. A study of patients with UC revealed that bifidobacteria populations are approximately 30 times lower than that of healthy individuals, indicating a dysbiosis that may potentially alter inflammatory responses [33,34]. However, it is unclear whether the change in flora is primary, or secondary to the disease. Diet has been shown to influence the balance of the intestinal flora [35]; therefore, it is conceivable that altering the diet can impact the inflammatory response [36].

Mucosal healing potential is another aspect of the IBD-AID [37]. Minimizing irritants, while increasing nutrient delivery in a phased manner, (see Table 2) according to patient symptomology is an important consideration to the diet. This is derived from the success of treatment of IBD with tube feeding as opposed to simply nothing by mouth (NPO) [38]. Nutritional deficiencies can result from reduced oral intake, malabsorption, medication side effects and systemic inflammation due to active disease [38,39]. In CD, the natural history of disease progression is a significant increase in complicated disease (from <29% patients at initial diagnosis having stricturing or penetrating disease to 88% of patients at 20 years) [40].

The Specific Carbohydrate Diet (SCD, from which the IBD-AID was derived and augmented) was developed by Dr. Sydney Valentine Haas and Dr. Merrill P Hass for the treatment of celiac disease in the mid-20^th^ century [41], and was later popularized as a treatment for IBD via the internet and lay population [42]. This nutritional regimen eliminates all grains, and encourages ingestion of probiotics in the form of homemade yogurt. The IBD-AID diet is not designed around avoidance of gluten, and strives to address other micro- and macronutrients not addressed in the SCD. The food supply, processing and transport of foods have changed dramatically since the inception of the SCD. The IBD-AID also modifies fatty acids, specifically decreasing the total and saturated fats and eliminating hydrogenated oils, and encouraging the increased intake of foods with omega-3 fatty acids. The IBD-AID also differs from the SCD with the inclusion of oats, since oats (and possibly other fermentable grains that provide a substrate for probiotics) [43] appear to be well tolerated and indeed are useful in regulation of bowel frequency and consistency [44].

The IBD-AID is a dietary pattern for which the mechanism and efficacy have yet to be elucidated. Poorly digested complex carbohydrates (even those with defined pre-biotic properties) may lead to bacterial overgrowth and bowel injury with increased intestinal permeability. The waste products of fermentation of undigested carbohydrates include methane, carbon dioxide, hydrogen, lactic and acetic acid, which are all gastrointestinal irritants. The carbohydrates allowed on the original diet are monosaccharides, that is, they have a molecular structure which allows intestinal absorption without additional enzymatic degradation. Improvement of nutritional status and alteration in ileo-colonic flora by certain foods is theorized to lead to decreased mucosal inflammation [2,45]. There has been an increase in publications over the past ten years with regard to food-based strategies used to alter the intestinal microflora as an alternative or adjunctive therapy for the treatment of IBD. The altered bacteria in the microbiome of IBD patients is said to drive inflammatory responses, [21,24] and there are a growing number of articles linking particular foods that influence the intestinal microbiome, by withholding certain foods that are not well tolerated by patients, or introducing others that can improve symptoms [46].

The dietary pattern of the IBD-AID is carefully oriented to decrease inflammation [14,15] and improve nutritional status, and thought to maintain a beneficial intestinal bacterial balance; however, this has yet to be proven by rigorous scientific examination. The current case series is promising, but must be followed with rigorous research and standardized study design to detect molecular markers of inflammatory and histo-pathological changes to correlate with clinical outcomes.

The IBD-AID can be difficult and restrictive diet; with adherence largely dictated by personal motivation, support from family and friends, and skills in the kitchen and the marketplace. Consultation and continued support from a knowledgeable dietitian may enhance compliance with the diet.

## Conclusion

Physicians are not provided with specific dietary treatments to offer their IBD patients [47], and recommendations for normal diet are often based upon a philosophy of “if it hurts, don’t do it”. This case series serves to highlight the importance of dietary manipulation as an adjunct to the limited existing management options for IBD. The study of the IBD-AID would benefit from the rigorous analysis provided by a randomized clinical trial, with evaluation of mucosal healing and assessment of change in gut flora to examine the exact mechanism(s) of benefit.

## Competing interests

The authors declare that they have no competing of interests.

## Authors’ contributions

The authors declare they have no competing interests. BO, TS, and DC conceived of the study, and saw the patients, and all authors helped to draft the manuscript. BO further developed the diet, and GP has been invaluable in the teaching kitchen, KB provided medical record extraction. YM provided statistical and epidemiological expertise to summarize results of this study. DC provided critical oversight and clinical supervision. All authors have read and approved the final manuscript.

## References

[B1] Day AS Ledder O Leach ST Lemberg DA Crohn’s and colitis in children and adolescents World J Gastroenterol 2012 18 5862 5869 10.3748/wjg.v18.i41.5862 23139601 PMC3491592

[B2] Brown AC Rampertab SD Mullin GE Existing dietary guidelines for Crohn’s disease and ulcerative colitis Expert Rev Gastroenterol Hepatol 2011 5 411 425 10.1586/egh.11.29 21651358

[B3] Balfour Sartor R Probiotics for Gastrointestinal Diseases UpToDate 2013 2013 UpToDate 2013.Location: 95 Sawyer Rd. Waltham, MA 02453

[B4] Bl AO Fecal Microbiota Tranplantation Curr Opin Gastroenterol 2013 29 1 79 84 10.1097/MOG.0b013e32835a4b3e 23041678

[B5] Roses RE Rombeau JL Recent trends in the surgical management of inflammatory bowel disease World J Gastroenterol 2008 14 408 412 10.3748/wjg.14.408 18200663 PMC2679129

[B6] Fazio VW Ziv Y Church JM Oakley JR Lavery IC Milsom JW Schroeder TK Ileal pouch-anal anastomoses complications and function in 1005 patients Ann Surg 1995 222 120 127 10.1097/00000658-199508000-00003 7639579 PMC1234769

[B7] Hahnloser D Pemberton JH Wolff BG Larson DR Crownhart BS Dozois RR Results at up to 20 years after ileal pouch-anal anastomosis for chronic ulcerative colitis Br J Surg 2007 94 333 340 10.1002/bjs.5464 17225210

[B8] Bassi A Dodd S Williamson P Bodger K Cost of illness of inflammatory bowel disease in the UK: a single centre retrospective study Gut 2004 53 1471 1478 10.1136/gut.2004.041616 15361497 PMC1774248

[B9] Carter MJ Lobo AJ Travis SP IBD Section, British Society of Gastroenterology Guidelines for the management of inflammatory bowel disease in adults Gut 2004 53 Suppl 5 V1 V16 15306569 10.1136/gut.2004.043372 PMC1867788

[B10] Yamamoto T Nutrition and diet in inflammatory bowel disease Curr Opin Gastroenterol 2013 29 216 221 10.1097/MOG.0b013e32835b9a40 23385526

[B11] Triggs CM Munday K Hu R Fraser AG Gearry RB Barclay ML Ferguson LR Dietary factors in chronic inflammation: food tolerances and intolerances of a New Zealand Caucasian Crohn’s disease population Mutat Res 2010 690 123 138 10.1016/j.mrfmmm.2010.01.020 20144628

[B12] Pearson M Teahon K Levi AJ Bjarnason I Food intolerance and Crohn’s disease Gut 1993 34 783 787 10.1136/gut.34.6.783 8314511 PMC1374262

[B13] Haas SV Haas MP Management of celiac disease 1951 Lippincott, Philadelphia

[B14] Shivappa N Steck SE Hurley TG Hussey JR Hebert JR Designing and developing a literature-derived, population-based dietary inflammatory index Public Health Nutr 2013 14 1 8 10.1017/S1368980013002115 PMC3925198 23941862

[B15] Cavicchia PP Steck SE Hurley TG Hussey JR Ma Y Ockene IS Hebert JR A new dietary inflammatory index predicts interval changes in serum high-sensitivity C-reactive protein J Nutr 2009 139 2365 2372 10.3945/jn.109.114025 19864399 PMC2777480

[B16] Lichtiger S Present DH Kornbluth A Gelernt I Bauer J Galler G Michelassi F Hanauer S Cyclosporine in severe ulcerative colitis refractory to steroid therapy N Engl J Med 1994 330 1841 1845 10.1056/NEJM199406303302601 8196726

[B17] Vermeire S Schreiber S Sandborn WJ Dubois C Rutgeerts P Correlation between the Crohn’s disease activity and Harvey-Bradshaw indices in assessing Crohn’s disease severity Clin Gastroenterol Hepatol 2010 8 357 363 10.1016/j.cgh.2010.01.001 20096379

[B18] Tamboli CP Neut C Desreumaux P Colombel JF Dysbiosis in inflammatory bowel disease Gut 2004 53 1 4 10.1136/gut.53.1.1 14684564 PMC1773911

[B19] O’Flaherty S Saulnier DM Pot B Versalovic J How can probiotics and prebiotics impact mucosal immunity? Gut Microbes 2010 1 293 300 10.4161/gmic.1.5.12924 21327037 PMC3023613

[B20] Fujimori S Gudis K Mitsui K Seo T Yonezawa M Tanaka S Tatsuguchi A Sakamoto C A randomized controlled trial on the efficacy of synbiotic versus probiotic or prebiotic treatment to improve the quality of life in patients with ulcerative colitis Nutrition (Burbank, Los Angeles County, Calif 2009 25 520 525 10.1016/j.nut.2008.11.017 19201576

[B21] Bosscher D Breynaert A Pieters L Hermans N Food-based strategies to modulate the composition of the intestinal microbiota and their associated health effects J Physiol Pharmacol 2009 60 Suppl 6 5 11 20224145

[B22] Jia W Whitehead RN Griffiths L Dawson C Waring RH Ramsden DB Hunter JO Cole JA Is the abundance of Faecalibacterium prausnitzii relevant to Crohn’s disease? FEMS microbiology letters 2010 310 138 144 10.1111/j.1574-6968.2010.02057.x 20695899 PMC2962807

[B23] McFarland LV Systematic review and meta-analysis of Saccharomyces boulardii in adult patients World J Gastroenterol 2010 16 18 2202 2222 10.3748/wjg.v16.i18.2202 20458757 PMC2868213

[B24] Furrie E Macfarlane S Kennedy A Cummings JH Walsh SV O’Neil DA Macfarlane GT Synbiotic therapy (Bifidobacterium longum/Synergy 1) initiates resolution of inflammation in patients with active ulcerative colitis: a randomised controlled pilot trial Gut 2005 54 242 249 10.1136/gut.2004.044834 15647189 PMC1774839

[B25] Lorea Baroja M Kirjavainen PV Hekmat S Reid G Anti-inflammatory effects of probiotic yogurt in inflammatory bowel disease patients Clin Exp Immunol 2007 149 470 479 10.1111/j.1365-2249.2007.03434.x 17590176 PMC2219330

[B26] Hou JK Abraham B El-Serag H Dietary intake and risk of developing inflammatory bowel disease: a systematic review of the literature Am J Gastroenterol 2011 106 563 573 10.1038/ajg.2011.44 21468064

[B27] Guerreiro CS Ferreira P Tavares L Santos PM Neves M Brito M Cravo M Fatty acids, IL6, and TNFalpha polymorphisms: an example of nutrigenetics in Crohn’s disease Am J Gastroenterol 2009 104 2241 2249 10.1038/ajg.2009.313 19550417

[B28] Triantafillidis JK Merikas E Georgopoulos F Current and emerging drugs for the treatment of inflammatory bowel disease Drug Des Devel Ther 2011 5 185 210 21552489 10.2147/DDDT.S11290 PMC3084301

[B29] Wall R Ross RP Fitzgerald GF Stanton C Fatty acids from fish: the anti-inflammatory potential of long-chain omega-3 fatty acids Nutrition reviews 2010 68 280 289 10.1111/j.1753-4887.2010.00287.x 20500789

[B30] Guijarro LG Mate J Gisbert JP Perez-Calle JL Marin-Jimenez I Arriaza E Olleros T Delgado M Castillejo MS Prieto-Merino D Gonzalez Lara V Pena AS N-acetyl-L-cysteine combined with mesalamine in the treatment of ulcerative colitis: randomized, placebo-controlled pilot study World J Gastroenterol 2008 14 2851 2857 10.3748/wjg.14.2851 18473409 PMC2710726

[B31] Choi JH Chung KM Park K Psychosocial predictors of four health-promoting behaviors for cancer prevention using the stage of change of Transtheoretical Model Psycho-Oncology 2013 29 22 2253 2261 10.1002/pon.3278 23630155

[B32] Podolsky DK The future of IBD treatment J Gastroenterol 2003 38 Suppl 15 63 66 12698875

[B33] Sheil B Shanahan F O’Mahony L Probiotic effects on inflammatory bowel disease J Nutr 2007 137 819S 824S 17311981 10.1093/jn/137.3.819S

[B34] Schwiertz A Jacobi M Frick JS Richter M Rusch K Kohler H Microbiota in pediatric inflammatory bowel disease J Pediatr 2010 157 240 244.e1 10.1016/j.jpeds.2010.02.046 20400104

[B35] Galland L Rakel: Integrative Medicine 2007 2 Philadelphia: Saunders Elsevier

[B36] Paturi G Mandimika T Butts CA Zhu S Roy NC McNabb WC Ansell J Influence of dietary blueberry and broccoli on cecal microbiota activity and colon morphology in mdr1a(-/-) mice, a model of inflammatory bowel diseases Nutrition 2012 28 3 324 330 10.1016/j.nut.2011.07.018 22113065

[B37] Osterman M Mucosal healing in inflammatory bowel disease J Clin Gastroenterol 2013 47 3 212 221 10.1097/MCG.0b013e3182732ff5 23340060

[B38] Massironi S Rossi R Cavalcoli F Della Valle S Fraquell IM Conte D Nutritional deficiencies in inflammatory bowel disease: Therapeutic approaches Clin Nutr 2013 S0261-5614 13 00098 8 10.1016/j.clnu.2013.03.020 23602613

[B39] Donnellan C Yann L Lal S Nutritional management of Crohn’s disease Therap Adv Gastroenterol 2013 6 3 231 242 10.1177/1756283X13477715 23634187 PMC3625021

[B40] Thia KT Sandborn WJ Harmsen WS Zinsmeister AR Loftus EV Jr Risk factors associated with progression to intestinal complications of Crohn’s disease in a population-based cohort Gastroenterology 2010 139 1147 1155 10.1053/j.gastro.2010.06.070 20637205 PMC2950117

[B41] Haas SV Haas MP The treatment of celiac disease with the specific carbohydrate diet; report on 191 additional cases Am J Gastroenterol 1955 23 344 360 14361377

[B42] Gottschall E Breaking the Vicious Cycle: Intestinal Health through Diet 2004 Baltimore, Ontario: Kirkton Press Ltd

[B43] Roberfroid M Prebiotics: the concept revisited J Nutr 2007 137 830S 837S 17311983 10.1093/jn/137.3.830S

[B44] Comino I Real A de Lorenzo L Cornell H Lopez-Casado MA Barro F Lorite P Torres MI Cebolla A Sousa C Diversity in oat potential immunogenicity: basis for the selection of oat varieties with no toxicity in coeliac disease Gut 2011 60 915 922 10.1136/gut.2010.225268 21317420 PMC3112367

[B45] Hardy H Harris J Lyon E Beal J Foey AD Probiotics, prebiotics and immunomodulation of gut mucosal defences: homeostasis and immunopathology Nutrients 2013 5 1869 1912 10.3390/nu5061869 23760057 PMC3725482

[B46] Tilg H Diet and intestinal immunity N Engl J Med 2012 366 181 183 10.1056/NEJMcibr1113158 22236230

[B47] Cohen AB Lee D Long MD Kappelman MD Martin CF Sandler RS Lewis JD Dietary patterns and self-reported associations of diet with symptoms of inflammatory bowel disease Dig Dis Sci 2013 58 1322 1328 10.1007/s10620-012-2373-3 22923336 PMC3552110

